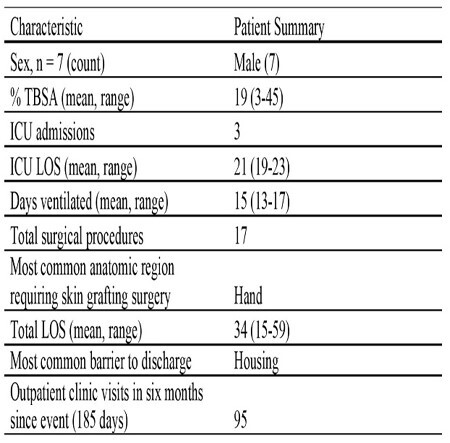# 541 In-Process Quality Improvement Action Following an Urban Residential Explosion

**DOI:** 10.1093/jbcr/irae036.175

**Published:** 2024-04-17

**Authors:** Emily Hagg, Danielle H Fuchko, Shyla K Bharadia, Vincent Gabriel

**Affiliations:** Foothills Medical Centre, Alberta Health Services, Calgary, AB; University of Calgary, Calgary, AB; Foothills Medical Centre, Alberta Health Services, Calgary, AB; University of Calgary, Calgary, AB; Foothills Medical Centre, Alberta Health Services, Calgary, AB; University of Calgary, Calgary, AB; Foothills Medical Centre, Alberta Health Services, Calgary, AB; University of Calgary, Calgary, AB

## Abstract

**Introduction:**

Burn centers need specialized plans for the management of burn mass casualty incidents for best outcomes. The ABA burn disaster response plan distributed in 2023 provides a communication plan for the association and burn centers. However, individual centers must develop local response plans. In this case, a Level 1 trauma and burn center with an institutional all-hazards, non-burn specific disaster plan responded to a home explosion in a city of 1.1 million. This event prompted a quality improvement project to assess the opportunities for development of burn-specific disaster response needs within the existing plan.

**Methods:**

The Define, Measure, Analyze, Improve, and Control (DMAIC) approach to quality improvement was utilized. Defining the problem and measuring current performance was conducted based on information derived from the electronic medical record system, Fire Department report and team debriefs of the incident response.

**Results:**

Seven male patients with a mean TBSA of 19% were admitted to the burn centre in a 1100 bed tertiary care hospital on a Monday morning. Three required ICU level care. The burn center had zero available beds at the time of incident and surge capacity was utilized. The total number of surgical procedures performed was 17. Mean length of stay was 34 days. There were 95 outpatient encounters in the six months following the incident (Table 1). All patients received specialized burn care and were discharged back to the community independent of basic activities of daily living. Increased human resources, equipment and consumable product use by physio and occupational therapy at the time of incident and in follow up was noted to be greater than anticipated. Social work needs exceeded capacity. On hand local specialized dressing supplies were exhausted within the first 24 hours and supplemental products were acquired from other centers and commercial vendors until available stock depleted. Strategies to improve initial communication have been implemented. Burn unit expansion is under discussion with the regional health authority. Stock levels and acquisition planning for supplies is underway. Additional physio, occupational therapy and social work needs require further measurement and analysis.

**Conclusions:**

Assessment of this center’s response to multiple simultaneous burn patient presentations offered an opportunity to optimize an existing all-hazards disaster response plan. Simulated burn disaster response is necessary to further assess the changes implemented and proposed.

**Applicability of Research to Practice:**

After event review may improve disaster response planning.